# The ChQoL questionnaire: an Italian translation with preliminary psychometric results for female oncological patients

**DOI:** 10.1186/1477-7525-8-106

**Published:** 2010-09-25

**Authors:** Giovanni Aschero, Flavio Fenoglio, Maria Giuseppina Vidili, Andrea Wussler

**Affiliations:** 1 Istituto Nazionale per la Ricerca sul Cancro, S.S. di Riabilitazione Oncologica, Viale Rosanna Benzi 10, I-16132 Genova, Italy; 2 Sacmar srl, Via Keplero 7, I-20019 Settimo Milanese, Italy

## Abstract

**Background:**

in Occidental languages, no widely accepted questionnaire is available which deals with health related quality of life from the specific point of view of Traditional Chinese Medicine (TCM). Some psychometric tools of this kind are available in Chinese. One of them is the Chinese Quality of Life questionnaire (ChQoL). It comprises 50 items, subdivided in 3 Domains and 13 Facets. The ChQoL was built from scratch on the basis of TCM theory. It is therefore specifically valuable for the TCM practitioner. This paper describes our translation into Italian of the ChQoL, its first application to Occidental oncological patients, and some of its psychometric properties.

**Methods:**

a translation scheme, originally inspired by the TRAPD procedure, is developed. This scheme focuses on comprehensibility and clinical usefulness more than on linguistic issues alone. The translated questionnaire is tested on a sample of 203 consecutive female patients with breast cancer. Shapiro-Wilk normality tests, Fligner-Killeen median tests, exploratory Two-step Cluster Analysis, and Tukey's test for non-additivity are applied to study the outcomes.

**Results:**

an Italian translation is proposed. It retains the TCM characteristics of the original ChQoL, it is intelligible to Occidental patients who have no previous knowledge of TCM, and it is useful for daily clinical practice. The score distribution is not Normal, and there are floor and ceiling effects. A Visual Analogue Scale is identified as a suitable choice. A 3-point Likert scale can also efficiently describe the data pattern. The original scales show non-additivity, but an Anscombe-Tukey transformation with γ = 1.5 recovers additivity at the Domain level. Additivity is enhanced if different γ are adopted for different Facets, except in one case.

**Conclusions:**

the translated questionnaire can be adopted both as a filing system based on TCM and as a source of outcomes for clinical trials. A Visual Analogue Scale is recommended, but a simpler 3-point Likert scale also suitably fits data. When estimating missing data, and when grouping items within Domain in order to build a summary Domain index, an Anscombe-Tukey transformation should be applied to the raw scores.

## Background

Traditional Chinese Medicine (TCM) has enjoyed a great deal of exposure in Occidental countries. As a consequence, there is an increasing need for psychometric tools specifically tailored to TCM. Tools developed in different medical contexts can of course be of use, but they are not necessarily optimal. The theoretical foundations of TCM are often unfamiliar to Occidental patients, so that Health Related Quality of Life (HRQoL) may be conceptualized differently by the TCM practitioner and the Occidental patient. On the one hand, quantitative psychometric tools are required to provide sound outcomes for clinical trials. On the other hand, the employment of generic tools, not specifically tailored to TCM, may result in insufficient sensitivity for those clinical trials. A standardized psychometric instrument based on TCM would be very useful, but at present no widely accepted generic questionnaire is available in Occidental languages.

In 2005 our Oncological Rehabilitation (O.R.) Unit started a data collection project, concerning acupuncture and TCM. On this basis, we later initiated a randomized clinical trial on the effectiveness of acupuncture treatments for breast cancer patients undergoing chemotherapy. Our aim was to ascertain whether acupuncture could relieve some of the side effects of chemotherapy. The generic EORTC QLQ-C30 questionnaire [1] and its related breast cancer specific module BR-23 were used in order to provide the main outcome, but the adoption of an additional questionnaire concerning HRQoL from the specific point of view of TCM was considered desirable.

The Chinese Quality of Life questionnaire (ChQoL) developed by Leung et al. [2-5] was identified as a possible option, due to its peculiarities with respect to the evaluation of acupuncture results. Being able to quantify HRQoL according to TCM was its explicit goal. The main characteristics of the ChQoL were its lack of specialization, its orientation to generic medicine, and the fact that it was highly structured. The questionnaire comprised 50 items, subdivided into 13 "Facets"; the Facets were grouped into 3 "Domains", namely "Physical Form", "Vitality & Spirit", "Emotion". Furthermore, this structure was built from scratch directly on TCM theoretical considerations, and then validated using Factor Analysis and Structural Equation Modeling [2,3].

The ChQoL was developed in Chinese. To our knowledge, no published translation is available in any Occidental language, except for a provisional "tentative" English translation reported in [2]. The present paper describes the translation procedure we adopted, the resulting Italian questionnaire, the score distribution in a sample of 203 patients, and some modifications to the response scales with respect to the original questionnaire. These modifications were deemed useful to adapt the ChQoL to the Italian cultural context. Some issues concerning internal consistency and additivity of scales are also considered. Our main interest at present is applicability to oncological patients. All the numerical results here reported concern a sample of female patients suffering from breast cancer.

## Methods

### Translation procedure

We adopted an iterative, multi-step, committee-based translation approach. Our procedure was initially inspired by the TRAPD framework ([6-8]; see also [9]). TRAPD is the acronym for five subsequent (but interrelated) phases: Translation, Review, Adjudication, Pre-testing and Documentation. This framework is particularly in use in social sciences, where cross-cultural differences are often an issue. However, the TRAPD original scheme was adapted and enlarged, so as to meet the specific needs of a TCM based instrument addressed to Occidental patients.

**Figure 1:**
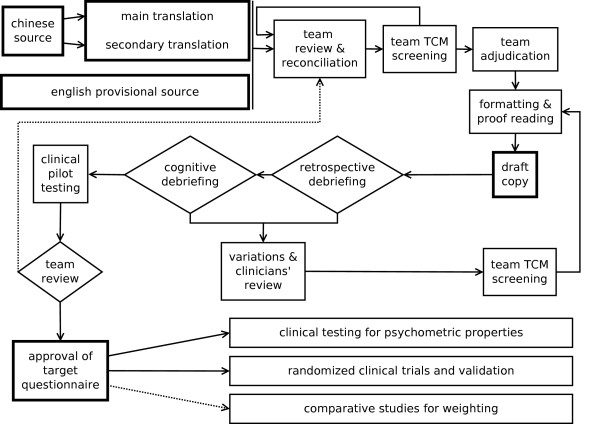
**Translation procedure**. Flow chart detailing the subsequent steps for translation. The dotted line represents a possible feedback path which, although originally considered, was ultimately found to be unnecessary.

Figure 1 shows a detailed flow chart of the translation procedure. Two separate translations were obtained, directly from the Chinese source. One was considered as "main" and one as "secondary". The two translators worked separately and independently. Both translators spoke mother tongue Italian, and had already received training in TCM at the time of translation. The first translator was a professional sinologist and interpreter, who had been residing in Beijing for several years. His work was intended to provide the best possible rendering of the original source into Italian, especially from the point of view of Conceptual and Semantic equivalence (we classify equivalence according to Herdman et al. see [10,11]). This was considered as the "main" translation. The second translator was a professional data analyst, with a background in questionnaire design and analysis. His task was more focused on disclosing issues regarding Operational and Measurement equivalence. This was considered as a "secondary" translation, to be used in suborder with respect to the first one.

A first series of meetings ("team review & reconciliation" in Figure 1) was held to review the two translations and the English source, and to reconcile them into a suitable Italian version. These meetings were attended by two medical doctors, the first translator, and the project coordinator (who was also the secondary translator). The two medical doctors were Italian acupuncturists, who had been studying and practicing TCM with patients for many years. Each component of the team was provided with the two translations and with the provisional "tentative" English version published by the original Chinese authors. After the team reached an agreement, a first reconciled Italian version was produced. At this stage, it was also decided to abandon the Likert scale adopted in the Chinese source, in favor of a Visual Analogue Scale (VAS). Therefore appropriate verbal descriptors were created, two for each line in the VAS. The reconciled version was further considered by the two medical doctors ("team TCM screening"), in order to screen adherence to TCM theory and to examine issues of comprehensibility on behalf of patients. Minor variations were proposed, and accepted by the team. A final meeting ("team adjudication") was held among the four components of the team, to agree on a final version. After formatting and proof-reading, a draft copy of the Italian version ("ChQoL-IT") was produced.

The draft copy was tested with a first round of retrospective debriefing interviews [12]. Eight volunteers received concise information about TCM and VAS, and then completed the questionnaire without supervision. Either a generic psychologist supervised by a medical doctor or a clinical psychologist alone reviewed the completed questionnaire together with each respondent, investigating missing data, problems of comprehension, and possibly offensive or problematic wording. Apart from these three issues, comments from the respondents were never solicited, but the interviewer was instructed to welcome any spontaneous comment.

The retrospective debriefing round was followed by cognitive debriefing interviews with 12 other volunteers. The questionnaire was completed without supervision. A medical doctor discussed the completed questionnaire with the respondent, on an item-by-item basis. The discussion aimed to detect if the original meaning had been correctly preserved in the translation, and if any unclear or ambiguous wording could generate misinterpretations. It was also specifically verified that the polarity of scales had been correctly recognized. The number of items was too high to discuss the entire questionnaire in one single interview. The questionnaire was divided into two parts, keeping either even or odd numbered items, and each volunteer was interviewed on one part only.

The results from the retrospective and cognitive interviews were analyzed by the project coordinator. On this basis, some variations concerning response scales and their verbal descriptors were proposed. The variations were reviewed by the two medical doctors ("variations & clinicians' review"), and after a new discussion concerning adherence to TCM theory ("team TCM screening") were approved by the team. After formatting and proof-reading, a new draft copy was finalized. An additional round of debriefing interviews was considered necessary, but it eventually yielded no further improvement.

The draft copy was therefore employed, without changes, to test clinical applicability ("clinical pilot testing" in Figure 1). The purpose was to ascertain differences between the patient's response and the doctor's opinion. The questionnaire was self-administered. Each response was then compared with what the doctor considered correct for that patient. Of course, this comparison was only possible for a few items, some issues being too personal to allow an external assessment. The full results are not part of this paper, and this topic will be covered in detail elsewhere. Preliminary results can be found in [13]. As far as it is of interest here, the comparison did not bring to light any specific bias which could advise against self-administration. The unattended modality of administration was consequently deemed valid for clinical use.

At a recapitulatory final meeting ("team review" in Figure 1) the team appraised the translated questionnaire according to four criteria: adherence to the original meaning, significance for TCM, clinical usefulness, and psychological impact on patients. The translation was considered satisfactory, and it was approved as the final version of the ChQoL-IT.

Three further actions were planned, as described in Figure 1: extensive clinical testing for psychometric properties, randomized clinical trials including validation, and comparative studies for weighting of scores in cross-cultural studies. The first has been accomplished, and its results will be described in the following paragraphs. A randomized clinical trial to evaluate the effects of acupuncture during chemotherapy has already been completed, and data analysis is in progress. The third action has been delayed, waiting for the full results from the randomized clinical trial.

### Clinical Testing

The questionnaire was handed to 230 consecutive patients. All patients were female, and had been recently diagnosed with breast cancer. All of them were undergoing, or were expected to undergo in a short time, conventional cancer treatment. No patient had previously received treatment with TCM at our Unit. The ChQoL-IT was self-administered, but prior to compilation each respondent was instructed on the questionnaire structure and aims, and on some aspects of TCM. The briefing was conducted by a medical doctor, and lasted less than 10 minutes. All the respondents were completing the questionnaire for the first time.

Of the 230 questionnaires, 27 had missing data and were not considered in the final sample. The reason is that, until data additivity has been either proved or recovered with proper techniques, handling of missing data is not straightforward. The usual linear techniques would not be applicable. Additivity will be considered in detail in the Discussion. Apart from this, no selection was made. The age of the 203 respondents ranged from 27 to 93 years, mean age ± SD was 57 ± 13 years, median age was 56 years. Only 106 out of 203 patients declared occupational status: 33% clerks and employees, 32.1% homemakers, 24.5% retired, 5.7% self-employed workers (professionals, managers, storekeepers, retailers), 3.8% manual workers, 0.9% unemployed. Data collection started on February 2006 and ended on September 2007.

This study was approved by the local Ethical Committee. Permission to conduct the study was obtained from the Head of the O.R. Unit. Written informed consent was obtained from the 20 participants in the debriefing interviews. No written informed consent was considered necessary for the 230 patients, because the ChQoL-IT just provided a rational, well organized modality to conduct the TCM examinations, identical to the examination the patient was currently undergoing. In fact, several questions in the ChQoL were already standard topics of those examinations. The adoption of the ChQoL-IT simplified the daily routine work, and it did not impose additional or unnecessary burden on patients.

### Data analysis

All scores were normalized to 0-100, the higher scores corresponding to a better health status. The score distribution was studied with Shapiro-Wilk normality tests and Fligner-Killeen median tests. Exploratory Two-step Cluster Analysis was also applied. The computation assumed an initial maximum of 15 clusters, a Bayesian information criterion for determining their number, noise handling at 25% for defining outliers, and minus log-likelihood for distance between clusters. The likelihood metric was preferred to the Euclidean because it resulted in a much lower number of outliers with our data. Scale additivity was examined by means of a Tukey's test for non-additivity (TTN) [14,15], including the Anscombe-Tukey power transformation. Calculations were performed using SPSS version 15 (SPSS Inc., Chicago IL) and the R statistical package version 2.7.2 (R Foundation for Statistical Computing).

## Results

### Target Questionnaire

The final target questionnaire ChQoL-IT is available in pdf format (Additional file 1). The 50 items are numbered progressively, grouped by Facet and Domain. The response scale is a VAS with horizontal lines, delimited at their extremities by short vertical lines, to avoid marking off the scale [16]. Lines have no gradations, to preserve sensitivity [17]. They are of equal length, and verbal descriptors are placed close to their extremities. For each item, the left side of the scale corresponds to a poor health status, whilst the right side corresponds to a better health.

### Clinical Testing

**Figure 2:**
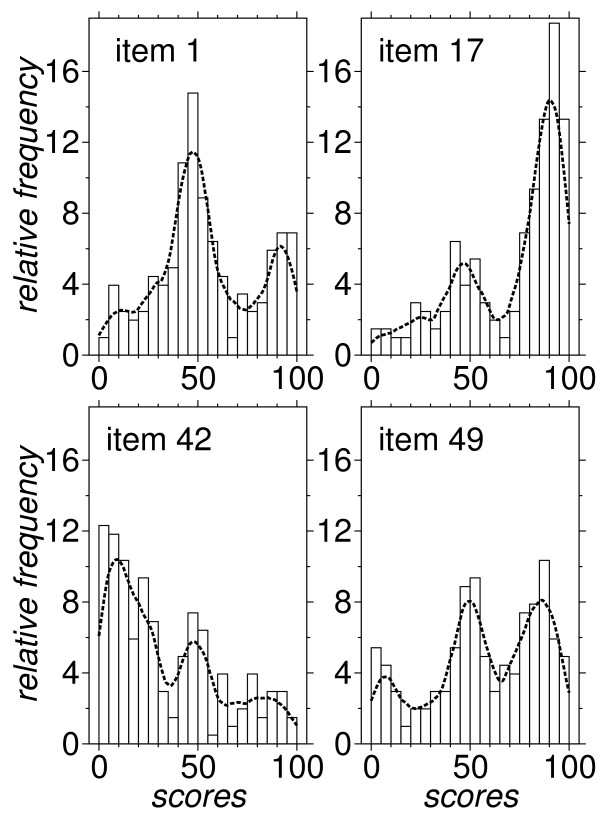
**Frequency distribution of scores for four items**. Relative frequency distribution of scores, expressed as percentage over the sample of the 203 respondents. Clockwise, starting from upper left: items 1, 17, 42, 49. The distribution for the other 46 items resembles one of these four cases. The dashed line is a smooth estimate obtained via an Epanechnikov kernel with bandwidth = 5.

Table 1 reports the scores for the sample of 203 respondents. Floor and ceiling effects are present, as shown by the high percentage of scores below 10 or above 90. A visual inspection of the frequency distributions confirms that a ceiling effect is present in approximately 60% of the items and a floor in 10% of them. Four examples are visible in Figure 2, which shows the frequency distribution for items 1, 17, 42, 49. These items have been selected because their distribution is representative. In fact, all the distributions show two, or even three, distinct peaks. The distribution around each peak is often truncated when the peak is near one end of the VAS.

**Table 1:** Score distribution

item	min	max	mean	median	floor	ceiling	skewness	Kurtosis
1	3	100	54.4 (25.4)	49.2 (39.3-75.0)	5%	14%	0.13	-0.74
2	2	100	48.1 (27.7)	46.6 (24.6-70.0)	11%	8%	0.11	-1.05
3	3	100	56.1 (25.4)	55.4 (38.3-76.8)	3%	11%	-0.12	-0.97
4	1	100	49.0 (29.7)	48.2 (24.8-76.7)	14%	11%	0.04	-1.22
5	2	100	54.4 (31.3)	50.0 (28.0-84.8)	10%	20%	-0.11	-1.35
6	1	100	57.7 (31.3)	60.9 (32.6-89.1)	10%	22%	-0.29	-1.33
7	1	100	53.9 (26.8)	50.0 (34.7-79.5)	5%	10%	-0.06	-1.01
8	0	100	49.8 (29.7)	47.9 (26.1-77.5)	14%	10%	0.00	-1.21
9	0	97	38.4 (28.5)	33.9 (10.2-60.2)	24%	5%	0.45	-0.99
10	0	98	44.5 (29.3)	43.2 (17.0-66.1)	15%	9%	0.25	-1.16
11	0	98	45.0 (29.6)	41.5 (19.5-73.9)	14%	9%	0.27	-1.22
12	1	100	47.2 (30.4)	44.9 (20.3-75.9)	14%	9%	0.11	-1.29
13	2	100	59.8 (30.6)	67.0 (33.3-88.4)	9%	22%	-0.40	-1.19
14	3	100	70.3 (26.3)	80.4 (48.2-91.5)	3%	31%	-0.94	-0.22
15	2	100	61.3 (29.8)	66.9 (36.4-89.3)	6%	23%	-0.47	-1.08
16	3	100	68.1 (26.3)	77.3 (47.5-91.1)	4%	28%	-0.77	-0.47
17	0	100	70.9 (26.3)	82.1 (50.0-91.5)	3%	32%	-0.92	-0.33
18	2	100	59.8 (29.0)	60.2 (35.7-87.6)	7%	21%	-0.37	-1.10
19	1	98	48.0 (29.7)	48.1 (19.6-72.9)	13%	13%	0.11	-1.25
20	2	100	60.3 (29.4)	66.1 (39.8-89.1)	7%	22%	-0.39	-1.12
21	4	100	71.7 (24.1)	79.5 (54.2-91.0)	3%	27%	-1.02	0.25
22	9	100	70.0 (24.3)	77.7 (50.0-91.1)	0%	30%	-0.76	-0.50
23	0	100	65.9 (25.2)	70.5 (48.3-88.4)	2%	22%	-0.61	-0.63
24	1	100	56.9 (27.3)	57.6 (38.3-80.4)	6%	14%	-0.32	-0.90
25	3	100	67.3 (25.2)	73.2 (48.2-89.0)	3%	21%	-0.74	-0.40
26	0	100	71.9 (22.8)	78.2 (56.3-90.7)	1%	27%	-0.91	0.01
27	0	100	71.1 (22.5)	77.1 (53.6-89.8)	1%	25%	-0.84	-0.07
28	4	100	69.6 (22.7)	76.3 (50.9-88.4)	0%	19%	-0.68	-0.51
29	4	100	67.0 (23.8)	70.5 (49.2-88.4)	1%	18%	-0.58	-0.57
30	4	100	71.0 (22.1)	76.8 (52.7-89.8)	1%	24%	-0.79	-0.12
31	2	100	73.3 (21.1)	79.8 (58.1-90.6)	1%	26%	-1.05	0.59
32	0	100	65.3 (24.7)	68.8 (48.3-87.5)	1%	19%	-0.58	-0.56
33	0	100	57.8 (26.4)	59.3 (42.9-80.5)	5%	14%	-0.31	-0.79
34	3	100	59.1 (26.0)	61.6 (43.2-79.5)	5%	13%	-0.42	-0.72
35	0	100	68.2 (25.9)	75.2 (49.1-89.9)	5%	25%	-0.88	-0.16
36	0	100	78.6 (20.5)	85.9 (70.3-93.2)	2%	37%	-1.67	2.78
37	0	100	55.7 (27.6)	55.1 (36.4-78.8)	8%	12%	-0.28	-0.91
38	2	100	62.7 (26.2)	66.7 (44.1-86.0)	5%	17%	-0.49	-0.67
39	2	100	59.9 (28.7)	61.2 (39.1-86.4)	8%	18%	-0.45	-0.94
40	3	100	76.1 (22.5)	84.7 (66.4-92.9)	1%	34%	-1.31	1.02
41	0	100	60.8 (27.2)	58.9 (46.4-85.9)	8%	19%	-0.51	-0.54
42	0	99	33.9 (27.5)	25.0 (10.2-50.0)	24%	4%	0.72	-0.59
43	0	100	49.3 (29.4)	49.1 (23.3-72.0)	13%	11%	-0.02	-1.17
44	2	100	69.2 (28.0)	79.7 (50.0-92.3)	5%	34%	-0.92	-0.29
45	0	100	63.4 (27.4)	68.8 (45.7-89.0)	5%	21%	-0.55	-0.77
46	1	100	60.0 (27.6)	63.6 (42.7-84.4)	7%	15%	-0.49	-0.80
47	0	100	59.5 (27.9)	62.7 (43.8-84.4)	6%	13%	-0.47	-0.88
48	2	100	65.6 (29.5)	76.5 (47.5-89.1)	9%	23%	-0.86	-0.52
49	0	100	57.7 (28.1)	55.4 (42.4-83.7)	10%	11%	-0.43	-0.85
50	2	100	58.2 (30.0)	63.3 (35.6-86.4)	9%	17%	-0.39	-1.15

For each item: minimum and maximum observed score (range is 0 - 100), mean with standard deviation, median with 25% and 75% percentiles, score floor and ceiling, skewness and kurtosis. Floor and ceiling are expressed as percentage of scores below 10 and above 90 respectively.

A Shapiro-Wilk test confirms absence of normality (p-value < 0.001 for each of the 50 items). Homogeneity of variances within Facet can be studied with a Fligner-Killeen median test, which is particularly robust against departures from normality [18]. The results are in Table 2; absence of homogeneity is evident in 7 out of 13 cases at a p-level of 0.05, notably for Facets Sleep, Verbal Expression, Joy, and Anger.

**Table 2:** Fligner-Killeen test

Domain	Facet	items	χ^2^	p-value
Physical	Complexion	4	10.5	0.01
	Sleep	3	15.9	0.00
	Stamina	6	3.4	0.49
	Appetite & Digestion	4	13.7	0.00
	Adaptation to climate	3	0.3	0.84

Vitality & Spirit	Consciousness	3	3.8	0.15
	Thinking	5	14.1	0.01
	Spirit of the eyes	2	2.8	0.09
	Verbal expression	2	13.0	0.00

Emotional	Joy	4	35.7	0.00
	Anger	5	24.1	0.00
	Depressed mood	6	2.9	0.72
	Fear & Anxiety	3	2.4	0.30

Fligner-Killeen median test for the homogeneity of variances The test is applied within Facet Dishomogeneity is found in 7 out of 13 Facets (p-level 0.05).

Table 3 reports the number of clusters identified by a Two-step Cluster Analysis. This kind of analysis automatically identifies an optimal number of clusters. The first subcolumn ("by item") pertains to a clustering applied item by item; the second ("by Facet") to a clustering where all the items within one Facet are considered at the same time. The latter analysis is legitimated by previously reported Factor Analysis results [2,3], which identify a single factor for each ChQoL Facet. Grouping into Facets tends to decrease the number of clusters, except for Facets "Appetite & Digestion" and "Spirit of the Eyes". This is a consequence of mixing information from different items. However, it is confirmed that a maximum of 3 clusters is always sufficient. Each cluster is identified by its centroid (mean and standard deviation) at the "by item" level. The number of cases which do not fit into the identified clusters is small, amounting to 3.9% in the worst case.

**Table 3:** Cluster Analysis

				Centroids							
						
		n. of clusters		cluster 1		cluster 2		cluster 3			
										
Facet	Item	by item	by Facet	mean	(sd)	mean	(sd)	mean	(sd)	outliers	extr mean
Complexion	1	3	2	21.0	(10.5)	50.6	(8.4)	89.5	(7.1)	--	55.3
	2	3	2	13.9	(7.8)	44.8	(7.8)	80.4	(11.3)	--	47.1
	3	2	2	35.6	(14.3)	-	-	79.3	(11.2)	--	57.5
	4	3	2	14.7	(9.5)	47.1	(7.8)	83.0	(10.2)	--	48.9

Sleep	5	2	2	29.4	(16.8)	-	-	85.2	(10.7)	--	57.3
	6	2	2	29.6	(17.1)	-	-	85.4	(10.0)	--	57.5
	7	2	2	36.7	(16.4)	-	-	83.8	(9.2)	--	60.3

Stamina	8	3	3	15.1	(10.1)	47.1	(7.3)	83.7	(9.5)	--	49.4
	9	2	3	15.8	(11.0)	-	-	65.6	(17.0)	--	40.7
	10	3	3	15.9	(9.8)	50.5	(8.7)	85.5	(8.0)	--	50.7
	11	2	3	25.7	(15.5)	-	-	79.4	(12.6)	--	52.6
	12	3	3	12.1	(7.9)	43.4	(8.8)	82.4	(10.2)	--	47.2
	13	2	3	29.1	(16.7)	-	-	83.7	(11.6)	--	56.4

Appetite	14	2	3	33.8	(15.9)	-	-	84.9	(10.9)	--	59.3
	15	2	3	36.7	(19.9)	-	-	87.6	(8.2)	--	62.1
	16	2	3	39.9	(17.0)	-	-	87.1	(8.1)	--	63.5
	17	2	3	38.6	(16.3)	-	-	88.2	(7.6)	--	63.4

Climate Adapt.	18	2	2	35.9	(18.1)	-	-	85.9	(9.2)	--	60.9
	19	3	2	16.7	(9.8)	54.5	(10.3)	88.5	(6.7)	--	52.6
	20	2	2	33.5	(17.1)	-	-	85.2	(10.0)	--	59.4

Consciousness	21	2	2	42.0	(17.1)	-	-	86.0	(9.2)	--	64.0
	22	2	2	48.5	(16.1)	-	-	88.8	(6.6)	1.5%	68.7
	23	2	2	51.0	(17.2)	-	-	89.6	(5.6)	3.0%	70.3

Thinking	24	2	2	36.1	(18.0)	-	-	81.6	(10.8)	--	58.8
	25	2	2	41.0	(16.8)	-	-	85.1	(9.3)	--	63.1
	26	2	2	54.0	(15.8)	-	-	89.4	(6.2)	2.0%	71.7
	27	2	2	45.3	(13.7)	-	-	85.4	(9.1)	0.5%	65.3
	28	2	2	44.6	(13.7)	-	-	85.5	(8.6)	--	65.1

Spirit	29	2	3	47.5	(15.0)	-	-	87.1	(7.6)	1.0%	67.3
	30	2	3	46.6	(14.3)	-	-	86.0	(8.6)	--	66.3

Verbal Expr.	31	2	2	52.3	(12.7)	-	-	87.0	(7.2)	2.0%	69.7
	32	2	2	46.1	(16.7)	-	-	87.2	(7.2)	0.5%	66.6

Joy	33	3	2	14.9	(8.7)	50.9	(9.6)	85.3	(8.5)	--	50.1
	34	3	2	18.7	(9.8)	53.6	(8.3)	83.2	(9.7)	--	51.0
	35	2	2	35.5	(16.8)	-	-	83.6	(10.6)	--	59.5
	36	2	2	64.4	(13.0)	-	-	91.5	(4.8)	3.9%	77.9

Anger	37	2	2	39.6	(19.8)	-	-	85.5	(8.4)	--	62.6
	38	2	2	46.8	(16.8)	-	-	87.8	(7.3)	3.0%	67.3
	39	2	2	36.0	(18.8)	-	-	84.9	(9.7)	--	60.5
	40	2	2	42.0	(16.0)	-	-	87.5	(8.4)	--	64.7
	41	3	2	12.2	(10.3)	52.3	(9.0)	87.9	(7.4)	--	50.0

Depression	42	2	2	14.6	(9.7)	-	-	63.5	(17.7)	--	39.1
	43	3	2	16.2	(10.8)	55.8	(10.6)	88.9	(6.7)	--	52.5
	44	2	2	35.8	(18.7)	-	-	87.1	(9.6)	--	61.5
	45	2	2	43.2	(19.3)	-	-	88.4	(7.2)	0.5%	65.8
	46	3	2	16.7	(10.4)	56.3	(10.6)	88.0	(6.8)	--	52.3
	47	2	2	34.4	(17.8)	-	-	82.1	(10.8)	--	58.2

Fear & Anx.	48	3	3	13.9	(10.0)	57.4	(10.3)	88.4	(6.5)	--	51.2
	49	3	3	12.8	(9.5)	49.5	(7.6)	84.1	(8.7)	--	48.5
	50	3	3	12.6	(6.9)	47.3	(8.1)	84.3	(9.7)	--	48.5

Optimal number of clusters identified by Two-step Cluster Analysis, applied either by item (columns 3) or by Facet (column 4). The centroids in the former case are reported, for each cluster. The number of outliers, if any, is expressed as percentage over the 203 respondents. The last column shows the mean of the external centroids (clusters 1 and 3).

This confirms that the clustering algorithm works properly with these data. The overall distribution of centroids is sharp for the intermediate and the right-end clusters (standard deviations 4.3 and 5.0 respectively). The spreading for the left-end cluster, which corresponds to a worse health status, is three times as much (standard deviation 13.9). The two outermost centroids are not equidistant from the half point of the VAS (score 50), their average half point being 58.4 (confidence interval at p = 0.95: 56.1-60.7). This means a slight shift towards a better health status. When the analysis is limited to the three clusters (15 cases), the intermediate cluster is centered on 50.8 (confidence interval at p = 0.95: 48.4-53.1), which is statistically compatible with the half point of the VAS.

Table 4 shows the results from a TTN. In 6 out of 13 Facets a lack of additivity is found. Some kinds of non-additivity can be removed by raising scores to a proper corrective factor γ (Anscombe-Tukey transformation). The three last columns in Table 4 show the TTN significance when three different γ are applied: the γ found applying the TTN by Facet; the γ found applying the TTN by Domain; and the mean of the γ found for the three Domains (γ = 1.5).

**Table 4:** Additivity and Tukey's correction factor γ

		untransformed scores			transformed scores		
			
Facet	n. of items	Friedman's χ^2^	p	γ	using γ by Facet	using γ by Domain	using constant γ = 1.5
Complexion	4	4.07	0.04	1.782	0.80	0.47	0.45
Sleep	3	0.81	0.37	2.090	0.71	0.93	0.91
Stamina	6	9.82	0.00	1.598	0.23	0.16	0.14
Appetite	4	0.39	0.54	1.327	0.80	0.94	0.93
Climate	3	0.21	0.64	1.143	0.88	0.59	0.61

Consciousness	3	1.23	0.27	1.737	0.55	0.59	0.44
Thinking	5	2.07	0.15	1.386	0.99	0.24	0.72
Spirit	2	3.01	0.09	2.401	0.49	0.29	0.20
Verbal Expr.	2	7.44	0.01	2.740	0.26	0.07	0.04

Joy	4	48.81	0.00	2.465	0.00	0.00	0.00
Anger	5	14.93	0.00	1.946	0.10	0.00	0.01
Depression	6	12.63	0.00	0.586	0.29	0.00	0.00
Fear & Anx.	3	0.56	0.46	0.665	0.65	0.39	0.27

Domain							
Physical form	20	13.81	0.00	1.522	0.35	0.35	0.30
Vitality & Sp.	12	9.60	0.00	1.811	0.83	0.83	0.26
Emotion	18	1.51	0.22	1.156	0.52	0.52	0.64

The Tukey's test for non-additivity is applied by Facet and by Domain, on the original untransformed score. Non-additivity is found in 6 out of 13 Facets and 2 out of 3 Domains (p-level 0.05). The three last columns show the p-level from the same test, but applied on scores transformed with different corrective factors γ. Third last column: uses γ from the previous column, same row; penultimate column: γ from the previous column, but by Domain (last three rows); last column: flat constant γ = 1.5.

## Discussion

### Target Questionnaire: Translation Procedure

Questionnaire translation can be dealt with by many different approaches, from the classical back-translation pioneered by Brislin forty years ago [19] to the more recent TRAPD procedure and its stems [6,9]. Different approaches are justified by different goals, so that the actual goals (and their priority) should always be declared *before *beginning the translation work. For a medical questionnaire, at least three main objectives can be identified: to preserve "equivalence"; to obtain a psychometric tool "useful" in the clinics and in clinical trials; and to attain full "comprehensibility" of the medical questions. Equivalence is what we commonly expect from a translation. What is really meant depends greatly on the researcher, so that Herdman et al. could identify not less than 19 different meanings for this term [10]. Clinical usefulness must be interpreted here as usefulness for the TCM practitioner. It includes using the questionnaire as a convenient filing system for anamnesis, but also providing a quantitative outcome for clinical trials. Comprehensibility is related both to the TCM theory and to the local cultural context. When a medical questionnaire is translated from a source to a target, the source and the target populations often share the same medical paradigms. When this happens, the three above mentioned objectives are likely not to interact with each other, or to interact minimally. As the medical theory is shared, the target and source populations also share a sort of common language.

In our case the situation is different. Not only do we have to cross the bridge between two totally different languages, we also have to face different medical paradigms. The main result is that our three objectives interact strongly. An excessive effort towards equivalence may be detrimental for comprehensibility. Each patient interprets questions on the basis of his or her cultural context. The risk is that an Occidental patient, when answering a TCM question, misinterprets it, and therefore does not provide what is actually useful for the TCM practitioner. These interaction mechanisms are at work in any translation, but may be particularly relevant here. Given the unfeasibility of reaching the three objectives at the same degree simultaneously, a choice of priorities must be made explicit. Of course, this choice influences the selection of the translation procedure.

Our first priority was clinical usefulness. Equivalence was of course a concern, but in suborder. Generally speaking, equivalence is desirable "for the cross-cultural comparison of results to be valid" [10]. The idea is that scores from different trials might be compared, for example in multicentre trials. As the questionnaire, conceived in a Chinese cultural context, was applied to Occidental patients, serious threats to equivalence were to be expected anyway. Therefore, we decided that giving priority to the equivalence issues would be inadvisable, whenever comprehensibility and clinical usefulness were at stake. This does not necessarily imply that equivalence is not ensured, but equivalence will have to be substantiated *a posteriori*. The specific case of operational equivalence is considered in the next section.

A modified TRAPD procedure was considered more suitable than a back-translation, in order to achieve our objectives. Weaknesses and inadequacies of back-translation have been summarized by Harkness et al. (see [20], page 468). Ponce et al. discuss some potential flaws of back-translation, and clearly warn that "translators have an incentive to choose word-for-word translations instead of striving for concept equivalence" [21].

The original Chinese version is written with clear and concise wording. This is due partly to the nature of the TCM lexicon, which rarely uses specialized words to designate syndromes, and partly to the original authors, who obviously made an effort to simplify questions. This is one of the reasons why we considered it safe to rely on one main translation only. In fact, the entire process up to the final version was not a direct, straightforward translation. It was a careful balancing of the linguistic issues, of the psychometric characteristics, and of the adaptation to the cultural (and medical) context. The main translation could have been the final version, but the secondary translation emphasized issues of measurement equivalence, and the team discussions delved more deeply into adherence to TCM theory. It is only the harmonious fusion of these three aspects what allowed a meaningful and useful final version. This attempt of fusion is the core of our translation, when compared with other procedures. Of course, we do not recommend our method for the general case. It would be unnecessarily burdensome and time-consuming. However, it proved to be efficient for the ChQoL. We suggest its use whenever the translation targets deeply different cultures, with very different medical contexts.

### Target Questionnaire: Response Scales

The response scale originally proposed for the ChQoL was a five-point Likert scale [2]. In this work, we intentionally adopted a VAS. Apart from a cautious consideration of the general advantages and disadvantages (a critical discussion of VAS can be found in [22-24]), our choice to depart from the original scale was motivated by four reasons.

First, we were particularly interested in the actual score distribution. Several items ask questions which, although perfectly intelligible, are rarely related to HRQoL in Occidental countries. For example, were the respondents able to utilize the entire continuous scale? And, if so, how widespread was this practice among respondents? Did they simplify their task assuming an essentially dichotomous model of good/poor health? A five-point Likert scale, which provides ordinal data, could in principle answer some of these questions, but a continuous scale was considered more suitable for our purpose.

Second, in the initial round of debriefing interviews we found some resistance to the five-point Likert scale. Several respondents found this scoring method unnatural, especially when the question concerned expressing emotions. The threat of annoyance is really important for our O.R. Unit, because of the poor health conditions and the high psychological reactivity of some patients.

Third, a VAS is known to be sensitive and reproducible [25-28]. It is widely used in oncology, even for multidimensional instruments [29]. In some cases, like pain assessment, a VAS is preferable to other kinds of scale, because it provides a closer description of the patients' experiences [30]. These characteristics are particularly useful in TCM clinical trials. TCM therapies may bring clinical results which, in the short term, are weaker than those brought by many pharmacological therapies. In these cases, a higher psychometric sensitivity is obviously of help.

Fourth, the respondents dealing with an analogue scale in a test-retest have less chance to recall their previous answers in order to show consistency [24]. Test-retest is an important aspect of reliability. Although we do not consider it in this paper, we are planning to investigate the problem in the future.

Our interpretation of the preference for the VAS among our patients is that evaluating our emotional status requires placing ourselves in a continuum. With the Likert scale, the respondent has to mentally adapt each of the 5 responses to an emotional status, and then decide if that answer "fits". The same question is likely to be re-read more times (possibly five, with really inattentive respondents). With the continuous VAS the respondent only has to spot the correct orientation of the scale regarding the question. The task requires less linguistic and comprehension efforts, and is more intuitive and straightforward. On the whole, it is less stressful.

This interpretation is founded on explicit feedback from the respondents during the first round of the retrospective debriefing interviews. One common comment was that joy, anger, depression or fear (items 33 to 50) are hardly quantifiable by ticking boxes. Other respondents felt "forced" into one of the five choices, which was unpleasant for them. However, results from other researchers contrast with our interpretation. Guyatt et al. [31] consider filling Likert scales more intuitive than selecting a position on a continuous line. Children and elderly people have been reported to prefer a Likert scale to a VAS, or to have problems understanding the VAS itself [32-35]. Gift reviews some difficulties reported for VAS [17]. Generally speaking, the preference for one scale towards another depends both on the scale and on the respondents. It is likely that different groups react in different ways. Our group was made of female oncological patients, and comparative studies with different groups could help clarify this point.

Another departure from the Chinese source lies in the orientation of the response scales. In the ChQoL-CN, 22 items out of 50 had a reverse (i.e. negative) polarity, the highest score corresponding to the poorest health status. Sometimes questionnaires are designed in such a way that polarity is reversed in approximately 50% of the items, in an attempt to force the respondent to pay more attention to the question, and avoid bias. This was not the original aim of the Chinese authors, as apparent from the distribution of the scales among Facets. In the ChQoL-CN, all items in Facets "Complexion" (4 items) and "Joy" (4 items), as well as in all the 4 Facets included in the "Vitality & Spirit" Domain (12 items), are positively oriented, whilst the Facets "Depression" (6 items) and "Fear" (3 items) show a reversed orientation. Obviously the developers' main goal was to optimize the response scale within the single Facet, whenever possible.

During the first round of debriefing interviews, it was found that the change in orientation from one item to another was confusing for many respondents and led to erroneous scoring. Consequently we decided to make all response scales conform to a positively oriented scale. This required the rephrasing of 22 questions. The second round of debriefing interviews showed no further problems concerning response scales.

### Target Questionnaire: Equivalence

Assessing questionnaire equivalence is not an easy task. A convenient framework for equivalence is provided by Herdman et al. [11]. These authors identify six key types of equivalence: Conceptual, Item, Semantic, Operational, Measurement, and Functional. An exhaustive discussion of equivalence for the two ChQoL versions must be deferred to another paper. This discussion would also require more experimental data. Nonetheless, there are a few points which can be discussed here. They may bring to light some limitations of the present work.

Operational equivalence is the main issue. This kind of equivalence refers to "the possibility of using a similar questionnaire format, instructions, mode of administration and measurement methods" [11]. Adopting a VAS instead of a 5-point Likert scale, and rewording several items to conform to a positively oriented scale does not necessarily mean that full Operational equivalence has been waived. A VAS and a 5-point Likert scale cannot be claimed to be equivalent, *a priori*. Hasson et al. show that a replacement of Likert scales with VAS is actually possible, but interchangeability is not necessarily ensured [36]. Lund et al. compare a VAS with a verbal rating scale, and find systematic disagreements when the VAS is transformed into a categorical scale [37].

Our adoption of a VAS was a trade-off between the full exploitation of the ChQoL psychometric potential for Italian patients and the aprioristic preservation of Operational equivalence. At this stage we are more interested in the former issue than in the latter. Our aim was to find a final version where the Italian patient would understand the significance of each question in exactly the same way as the Chinese patient. Within Herdman's framework, we tried to favor Conceptual and above all Semantic equivalence. Conceptual equivalence ensures that questions have "the same relationship to the underlying concept in both cultures", whilst Semantic equivalence "is concerned with the transfer of meaning across languages, and with achieving a similar effect on respondents in different languages" [11]. Our choice for a VAS and for a positive orientation of items was based on our relational experience with our patients, but it was particularly guided by the quotation above, regarding Semantic equivalence.

Our conclusions are founded on a specific sample. First of all, our respondents were Occidental patients. We by no means suggest that our choices are optimal for other cultures. E.g., Wong et al. [5] studied the validity of the ChQoL in Hong Kong. In that context, it would have made no sense for Wong and colleagues to adopt our (or similar) choices for the response scales. These choices are useful for the Italian cultural context, but they may be totally unnecessary in different cultures. Secondly, our sample is made up of female oncological patients, with a recent breast cancer diagnosis. We selected this sample because we deal with this kind of patient on a daily basis. Of course this sample is not generic, and it has peculiar characteristics. These patients may show heavy emotional and psychological suffering. They also may experience postural problems and limb disability. In a sense, our sample was a sort of "worst case benchmark" for the ChQoL. Our finding is that the ChQoL is robust enough to be applicable to this kind of patient, provided that some modifications to the response scale are implemented. Future research may find that no advantage is gained from the modified response scales, whenever the sample comprises generic patients only. The numerical results in Table 1 should not be taken as a norm for generic populations. Our opinion is that equivalence (as a whole) can be preserved more with our changes to the response scales than without. A literal translation is not necessarily faithful, as it may not preserve Semantic equivalence. We may be mistaken. However, an experimental comparison of our results (on a wider and more generic sample) with those obtained from a Chinese cultural context is necessary to solve this issue. Until this comparison is completed, full equivalence between the ChQoL-IT and the ChQoL-CN cannot be claimed, and the ChQoL-IT should not be used for cross-cultural comparative studies.

### Clinical testing: Scores and Distribution

The raw scores are not normally distributed. The choice of a VAS instead of a Likert scale efficiently highlights this point. The ChQoL capability to reveal floor and ceiling effects is in fact of great psychometric interest. These effects may reflect the presence of psychological resistances. Being able to unveil these resistances is particularly useful for the clinical psychologist dealing with frail patients, as oncological patients often are.

Severe deviations from normality, including skewness and ceiling effects, are not uncommon in Patient Reported Outcomes (e.g. [38,39]). Usually they can be reduced by modifying the response scales (e.g. [40,41]). However, more than just simple skewness, here we deal with a multimodal distribution. All shapes in Figure 2 are consistent with a superimposition of two or three bell-shaped curves. The Two-step Cluster Analysis confirms that the score distribution for 35 items is optimally split up into 2 clusters, and for 15 items into 3. One possible interpretation is that the respondents of the ChQoL are faced with unusual questions, which to them are seemingly unrelated to HRQoL. As a result, the respondents tend to simplify their task, dichotomizing their responses as being "yes or no". Some of the items generate more indecision than others, so that a third intermediate peak is possibly found in the score distribution. As a whole, a three-peak model seems reasonable for all items, with a chance that one (or even two) peaks turn out to be too low to be detected. If this model holds, a 3-point Likert scale is naturally suggested. Its adoption would make the ChQoL nimbler, and simplify data collection. It would also make localization in other languages easier. A drawback could be reduced sensitivity to changes, or reduced discriminability among individuals. It has been reported that placebo effects may sometimes remain undetected when using strictly binary outcomes, whilst they are detected when using continuous outcomes [42].

### Clinical testing: Additivity

In a Tukey's test, additivity is conceived as a lack of interaction between the respondents and the items, in the framework of a linear model. Additivity is a desirable property, because it simplifies handling of missing data and development of concise indexes to sum up a scale. The main result from Table 4 is that almost one half of the Facets ("Complexion" and "Stamina"; "Verbal Expression"; "Joy", "Anger" and "Depression") shows evidence of non-additivity. This suggests that using raw ChQoL data as outcomes in a clinical trial is not advisable. Raw scores should undergo some kind of pre-analysis correction. A corrective factor γ is provided by the TTN itself. Its aim is to yield additivity. When an Anscombe-Tukey transformation is applied, i.e. when all scores within one Facet are raised to γ, additivity is achieved for all Facets but "Joy". In fact, the transformation suggested by the TTN is not necessarily helpful for reducing non-additivity. The TTN assumes a quadratic model for the hypothetical respondent-by-item interaction. If the actual kind of non-additivity is different, the Anscombe-Tukey transformation may be ineffective. We can infer that there is a complex kind of respondent-by-item interaction for this Facet. Additivity can be achieved at *p *≥ 0.05 using γ ≃ 3.9, but of course such a high γ heavily distorts the frequency distribution, and it is not of any practical use. It should be noted that Facet "Joy" belongs to the Emotional Domain: evaluating and expressing emotions is not an easy task, and a strong respondent-by-item interaction is more plausible than for the other two Domains.

Obtaining additivity for 12 out of 13 Facets is a satisfactory result. Nonetheless the γ values are tailored to our specific sample, and they are all different. In order to make practical applications easier, we explored two alternatives. The first is to compute γ at the Domain level, and not at the Facet level. Then the γ can be applied either to the pool of items within one Facet or to the pool of items within one Domain, and the TTN can be run again. When applied within Facet, problems are again encountered for Facet "Joy", and additionally for "Anger" and "Depression" too. All these Facets belong to the Emotional Domain. When applied within Domain (γ in Table 4, penultimate column, three last rows), full additivity is finally achieved. The second alternative is to try a flat γ = 1.5 (which is the mean of the three γ found for the three Domains). When proceeding by Facet, the statistical significance is too low (*p *< 0.05) for three Facets in the Emotional Domain, and also for one Facet in the Vitality & Spirit Domain. When proceeding by Domain, the test significance is acceptable, although for the Vitality & Spirit Domain it is lower than before (*p *= 0.26 versus *p *= 0.83).

Altogether, a constant γ = 1.5 seems a practical choice. It provides additivity at the Domain level, which is our main interest. It is low enough not to excessively distort data. Given Table 4, it lies inside a full range of acceptable γ, so increasing the chances of applicability to different populations of patients. We suggest that an Anscombe-Tukey transformation with γ = 1.5 is applied to the ChQoL-IT scores whenever the scores are used to estimate missing data or to provide summary indexes for the three Domains. This (or an equivalent) kind of transformation has been shown to be necessary for our specific sample of female patients suffering from breast cancer, in order to recover additivity. When dealing with different populations, the necessity and advisability of a γ = 1.5 transformation should of course be checked.

## Conclusions

The applicability of TCM questionnaires to Occidental patients should not be taken for granted, as cultural differences may play a decisive role. Clinical usefulness, comprehensibility, and adherence to the original source represent important but possibly competing requirements. We propose a customized translation procedure in order to meet these requirements. The resulting Italian version of the ChQoL questionnaire has proven to be comprehensible and meaningful for the Occidental layperson, and applicable to a sample of female oncological patients suffering from breast cancer. Scales for this sample show evidence of non-additivity, but additivity is recoverable with a simple γ = 1.5 Anscombe-Tukey transformation. The tasks of estimating missing data and of constructing summary scores are consequently simplified. The translated questionnaire can therefore be adopted in clinical trials, to provide quantitative outcomes for TCM.

Our results are based on a VAS, but we show that a simpler 3-point Likert scale also provides a good description of data. In both cases, we relinquish Operational equivalence regarding the original source questionnaire, which uses a 5-point Likert scale. More research is needed to fully assess the five other types of equivalence, and also to shed light on certain reliability issues.

## Abbreviations

ChQoL: Chinese Quality of Life questionnaire; TCM: Traditional Chinese Medicine; HRQoL: Health Related Quality of Life; O.R.: Oncological Rehabilitation; VAS: Visual Analogue Scale; TTN: Tukey's Test for Non-additivity.

## Competing interests

The authors declare that they have no competing interests.

## Authors' contributions

All authors participated in each of the team discussions. FF and MGV led the clinical discussions, conducted or supervised the interviews and the debriefing sessions, and managed all the interactions with patients. AW provided the main translation, led the reconciliation and adjudication meetings, and took care of the linguistic aspects. GA conceived of the study, provided the secondary translation, supplied data analysis, and coordinated the team. All authors contributed to drafting the manuscript. All authors read and approved the final manuscript.

## Supplementary Material

Additional file 1 **Final Italian version of the ChQoL**. Click here for file
