# Supplementary material for: The ChQoL questionnaire: an Italian translation with preliminary psychometric results for female oncological patients
Source: Health Qual Life Outcomes. 2010 Sep 25;8:106. doi: 10.1186/1477-7525-8-106 (PMC2958157; doi:10.1186/1477-7525-8-106)
Supplement: Additional file 1 — Final Italian version of the ChQoL. [file 1477-7525-8-106-S1.PDF]

Questionario ChQoL-IT

Qualità della vita secondo la Medicina Tradizionale Cinese

Aspetti fisici e somatici

1.1 Aspetto fisico

01. Come ritiene che sia il colorito del suo viso?

Colorito pessimo |———| *Colorito ottimo*

02. Ritiene che il suo viso sia luminoso?

No, pochissimo |———| *Sì, moltissimo*

03. Come ritiene sia il colorito delle sue labbra?

Colorito pessimo |———| *Colorito ottimo*

04. Ritiene che le sue labbra siano ben idratate?

No, per niente |———| *Sì, moltissimo*

1.2 Sonno e riposo

05. Soffre di insonnia?

Sì, moltissimo |———| *No, per niente*

06. Sognare disturba la qualità del suo riposo?

Sì, moltissimo |———| *No, per niente*

07. Come dorme?

Malissimo |———| *Benissimo*

1.3 Vigore e forza vitale

08. Cammina con passo leggero?

No, per niente leggero |————————| *Sì, assai leggero*

09. Si stanca facilmente?

Sì, moltissimo |————————| *No, per niente*

10. Manca di forza nelle braccia e nelle gambe?

Sì, manco moltissimo di forza |————————| *No, non manco per niente di forza*

11. Si sente vigoroso?

No, niente affatto vigoroso |————————| *Sì, assai vigoroso*

12. Ha spesso la sensazione che le sue forze la tradiscano?

Sì, costantemente |————————| *No, mai*

13. Soffre facilmente di difficoltà di respiro (cioè di "dispnea")?

Sì, ne soffro assai facilmente |————————| *No, non ne soffro affatto*

1.4 Appetito e digestione

14. Trova il cibo gustoso?

No, per niente gustoso |————————| *Sì, assai gustoso*

15. Le sembra di avere problemi di digestione?

Sì, ho una digestione pessima |————————| *No, ho una digestione ottima*

16. La quantità di cibo che mangia è normale?

No (troppa, o troppo poca) |———| *Sì, del tutto normale*

17. Com'è il suo appetito?

Non normale (assente, oppure esagerato) |———| *Del tutto normale*

1.5 Adattabilità al clima

18. Riesce ad adattarsi bene ai cambiamenti di stagione e di clima?

No, ci riesco malissimo |———| *Sì, ci riesco benissimo*

19. Il suo corpo risente dei cambiamenti di stagione e di clima?

Sì, ne risente sempre moltissimo |———| *No, non ne risente mai*

20. Il suo corpo risente negativamente dei cambiamenti di orario durante la giornata?

Sì, ne risente negativamente |———| *No, non ne risente negativamente*

Aspetti mentali e cognitivi

2.1 Coscienza e consapevolezza

21. Ritiene di avere una mente lucida?

No, per niente lucida |———| *Sì, completamente lucida*

22. E' in grado di reagire al mondo esterno in maniera appropriata?

No, non sono affatto in grado |———| *Sì, sono perfettamente in grado*

23. Riesce a concentrarsi?

No, non ci riesco affatto	|—————————|	*Sì, ci riesco benissimo*

2.2 Pensiero e memoria

24. Ha buone capacità di memoria?

No, ho una pessima memoria	|—————————|	*Sì, ho un'ottima memoria*

25. Le sue reazioni sono pronte?

No, non sono per niente pronte	|—————————|	*Sì, sono assai pronte*

26. Quando riflette su un problema ragiona chiaramente?

No, non ragiono chiaramente	|—————————|	*Sì, ragiono chiaramente*

27. Quando riflette riesce a mantenere la sua capacità di attenzione?

No, non ci riesco affatto	|—————————|	*Sì, ci riesco benissimo*

28. Il suo pensiero è agile, capace?

No, per niente	|—————————|	*Sì, assai*

2.3 Occhi e sguardo

29. Ha uno sguardo vivace?

No, niente affatto vivace	|—————————|	*Sì, assai vivace*

30. I suoi occhi sono espressivi?

No, pochissimo	|—————————|	*Sì, moltissimo*

2.4 Capacità espressive verbali

31. La sua voce è chiara quando parla?

No, pochissimo |———| *Sì, moltissimo*

32. Riesce a trovare le parole adeguate per esprimere le sue idee?

No, non ci riesco mai |———| *Sì, ci riesco sempre*

Aspetti psicologici ed emozionali

3.1 Gioia di vivere

33. Si considera una persona contenta?

No, per nulla |———| *Sì, del tutto*

34. E' soddisfatto della vita?

No, per nulla |———| *Sì, del tutto*

35. Si considera una persona serena, in pace con sé stessa?

No, per nulla |———| *Sì, del tutto*

36. Ha interesse per la vita?

No, pochissimo |———| *Sì, moltissimo*

3.2 Rabbia, irritazione

37. Si infastidisce facilmente?

Sì, moltissimo |———| *No, pochissimo*

38. E' irascibile?

Sì, moltissimo ├────────┤ *No, per niente*

39. Ha facilmente un comportamento agitato e impaziente?

Sì, assai facilmente ├────────┤ *No, assai difficilmente*

40. Ha spesso un comportamento impetuoso o violento?

Sì, assai spesso ├────────┤ *No, mai*

41. E' in grado di controllare il suo stato emotivo?

No, per niente ├────────┤ *Sì, perfettamente*

3.3 Tendenze depressive

42. Tende a farsi carico emotivamente di qualsiasi cosa?

Sì, moltissimo ├────────┤ *No, pochissimo*

43. Si sente spesso triste?

Sì, sempre ├────────┤ *No, mai*

44. Si sente spesso disperato e senza aiuto?

Sì, sempre ├────────┤ *No, mai*

45. Si sente spesso oppresso, soffocato, impossibilitato a provare piacere nelle cose?

Sì, sempre ├────────┤ *No, mai*

46. Ha frequentemente voglia di piangere?

Sì, sempre ├────────┤ *No, mai*

47. Si sente spesso depresso?

Sì, sempre |———| *No, mai*

3.4 Paure e ansietà

48. Ha frequentemente paure senza alcun valido motivo?

Sì, sempre |———| *No, mai*

49. Prova spesso una sensazione di insicurezza?

Sì, sempre |———| *No, mai*

50. Si spaventa facilmente?

Sì, sempre |———| *No, mai*

Grazie per aver compilato il questionario.
